# The Study of Pentoxifylline Drug Effects on Renal Apoptosis and BCL-2 Gene Expression Changes Following Ischemic Reperfusion Injury in Rat

**Published:** 2014

**Authors:** Mehrdad Hashemi

**Affiliations:** *Department of Pharmacology and Toxicology Pharmaceutical Sciences Branch Islamic Azad University, Tehran, Iran.*

**Keywords:** Pentoxyfylline, Ischemia, BCL-2 gene, Real-Time PCR

## Abstract

Ischemia Reperfusion injury is the tissue damage caused when blood supply returns to the tissue after a period of ischemia or lack of oxygen. In this study, the effect of pentoxyfylline on BCL-2 gene expression changes and cell injury in kidney of rat following Ischemia Reperfusion were evaluated.

In this experimental study, 20 male wistar rats with average weight of 250-300 g were selected and then were accidently divided them on two tenth group of control and treatment groups. In the control group, celiotomy was performed by ventral midline incision. The left kidney was isolated, and then both the renal artery and vein were obstructed. After 60 minutes of warm ischemia, vessel obstruction resolved and the right kidney was removed. 72 hours after reperfusion, tissue samples were taken from left kidney for Tunel assay. We used quantitative real time PCR for detection of BCL-2 gene expression in treated groups and then compared them to control samples.

In the treatment group, the cell death changes, showed lower level than the control group. The results also showed the BCL-2 gene expression was declined in ischemia group as campared to PNT drug group.

The pentoxyfylline might have a role in control of apoptosis result from Ischemia- reperfusion and quantitative real-time PCR can be used as a direct method for detection BCL-2 gene expression in tested samples and normal samples.

## Introduction

Ischemia-Reperfusion is a kind of complex clinical syndrome that in particular situation it might impress different body’s organs, as it is apparent from its name it has one level of reduction or obstruction of perfusion to the tissue and after a while reperfusion to that organ (1). Ischemia Reperfusion injury is the tissue damage caused when blood supply returns to the tissue after a period of ischemia or lack of oxygen (2, 3). The absence of oxygen and nutrients from blood during the ischemic period creates a condition in which the restoration of circulation results in inflammation and oxidative damage through the induction of oxidative stress rather than restoration of normal function. Oxidative stress derived from IR injury through producing the reactive oxygen species and oxidative damage causes DNA modification, lipid peroxidation and secretion of inflammatory cytokines. This process continued and moves on the proteolytic events and induces the apoptosis. Plenty of proteins and number of genes regulate the apoptosis, in which two pairs of proteins are more important. BCL-2 family of proteins is found in external surface of mitochondria and divided to 3 groups including: antiapoptotic proteins like BCL-2 and BclxL, proapoptotic proteins like Bax and BAD and the proteins with apoptotic activity like Bik .After occurring a severe damage in the DNA of cells in a way that cannot be repaired, it will undergo the apoptosis in which activation of p53 causes proapoptotic protein. Bax protein exists in the external membrane of mitochondria with some other proteins making the complex of Apoptosome, which has the key role in activating the caspases and inducting the apoptosis (4).We selected BCL-2 gene, because BCL-2 family of proteins functions as pro- and anti-apoptotic members.This process continued and moves on the proteolytic events and induces the apoptosis.

The inflammatory response partially mediates the damage of reperfusion injury. The inflammatory response partially mediates the damage of reperfusion injury. White blood cells carried to the area by the newly returning blood, release inflammatory factors such as interleukins and tumor necrosis factor (TNF-α) (5,6). (TNF)-α stimulates the production of BCL-2 family member protein from the cytoplasm to the outer mitochondrial membrane (7, 8, 9 ). This issue causes mitochondrial swelling and induces apoptosis (10) Therefore, pharmacological agents could decrease the production of TNF-α in process of ischemia-reperfusion injury that results in reduced reperfusion injury (11-17). Pentoxifylline (PTX) is a drug that has multiple properties. It decreases oxygen and the production of free radicals (16) and inhibits TNF-α in mononuclear phagocytes. A study indicated that PTX has some protecting effects on remote kidney injury only in the early phase of reperfusion due to ischemia-reperfusion injury (17), but another study indicated that PTX decreases oxidative damage in rat liver after ischemia-reperfusion (18). In study indicated that the PTX has some protecting effect on remote kidney injury dueto ischemia / reperfusion injury only in the early phase of reperfusion (19). In study indicated PTX decreased oxidative injury in rat liver after ischemia reperfusion (20). In this study, the role of pentoxifylline drug in renal cells death function following ischemia-reperfusion was examined and we have designed and optimized quantitative real-time PCR assays based on SYBR Green I chemistry for determining the PTX drug effect on BCL-2 gene expression changes.

## Experimental

*Animals*

In this experimental study, 20 adult wistar rats were selected and healthily function of renal of these animals was determined by measuring creatinine and blood urea nitrogen. Rats were randomly allocated into two control group (n = 10) and treatment with pantoxifylline group (n = 10). In control group,for inducing anesthesia about 12 mg/Kg Ketamine intraperitoneal by injection method was used. Celiotomy was performed by ventral midline incision the left kidney was isolated, and then both the renal artery and vein were obstructed. After 60 minutes ischemia, the vessels opened and reperfusion was performed. Then the right kidney was removed by using the nephrectomy. The time of blood reperfusion in this study was 3 days. After surgery, the animals were allowed to have free access to water. In treatment group with pantoxifylline, all the actions above were done except that this group had used pantoxifylline dose of 45 mg/Kg orally 3 hours before surgery. This treatment was continued once every 12 hours for 3 days. For microscopic studies, at the end of experiment and 72 hours after reperfusion, left kidney of rats separated by dislocation in neck vertebra and stabilized in 10% buffer formalin and then was sent to the pathologic laboratory. Of left ventricle of rats’ heart, consecutive sections with a thickness of 5 microns were prepared for TUNEL specific staining and histopathologic sections were evaluated by optical microscope Nikon model (ECLIPSE E200, made in Japan). The cells that were positive in apoptosis occurrence were counted and analyzed by using light microscopy.

*Tunel assay*

For counting of apoptotic cells, five microscopic fields in each section were randomly selected from the left ventricle and were investigated with x40 magnification. Diagnostic technique apoptosis according to TUNEL kit instruction (Insitu cell death, detection kit, POD, Roche Company, made in Germany) was performed. In summary, the slices were deparaffinized and dehydrated, and then were washed in distilled water. Tissues were stabled for 15 minutes at room temperature with 20 g/mL K protein kinase (Boehringer Mannheim, Mannheim, Germany). Endogenous peroxidase activity was blocked by the incubation in 3 mL/L of hydrogen peroxide/methanol for 30 minutes at 37 °C. Slices were incubated with terminal deoxynucleotidiltransferase for 60 minutes at 37 °C. Then dUTP (Deoxyuridine triphosphate) conjugated molecules with deoxygenine were added to the end of 3-OH frogmanthe DNA. The antibody anti-deoxygenine peroxidase was used for diagnosis marked nucleotides. Slices were stained by DAB (Diaminobenzidine) and Hematoxylin was used for background staining (10). 

*RNA isolation and reverse transcription*

The tissue samples were treated with total RNA isolation reagent (Sigma) as recommended by the manufacturer and the extracted RNA was purified using RNeasy Mini Kit (Qiagen). The concentration and purity of the purified RNA were determined by spectrophotometry. High quality RNAs (A260/280 ≥ 1.8) were selected and kept at -80 °C until use for cDNA synthesis. Up to 1 μg RNA was converted to cDNA using QuanTitect® Reverse Transcription Kit (Qiagen) according to the manufacturer's instruction. To verify the integrity of the cDNA, a PCR experiment was performed using glyceraldehydes-3-phosphate dehydrogenase (GAPDH) specific primer. The primers for real-time PCR of BCL-2 and *GAPDH* genes were designed by the Primer Express v.3.0 software (Applied Biosystems, Foster City, USA).

*Real-time PCR with SYBR green I*

The selected primers were underwent an extensive search using BLAST tool (www.ncbi.nlm.nih.gov/blast) .The characteristics of the primers used in this study have been summarized in Table 1. Real-time PCR was carried out in optical grade 96-well plates (Micro amp, Applied Bio systems, Singapore) at reaction volume of 25 mL, including 12.5 SYBR Green Master Mix (primer design), 300 nM primer and 5 ng template DNA. All samples were run in duplicate. Thermal cycling was performed on the Applied Biosystems 7300 real-time PCR system using the following cycling conditions: 95 °C for 10 min, and 40 cycles at 95 °C for 15 s, and 60 °C for 1 min. Each complete amplification stage was followed by a dissociation stage at 95 °C for 15 s and 60 °C for 30 s. Then, temperature was ramped up from 60 °C to 95 °C ( 0.03 °C/s), and fluorescence intensity data was collected continuously over the ramping stage for 20 min. Melting curve analysis was performed according to the dissociation stage data and reactions with a single peak at expected temperature melting (Tm) were considered for further analysis.

**Table 1:** Characteristics of the primers used in the real-time PCR assay

**Gene**	**Sequence**	**Tm**	**product length**
rat-BCL-2-F	ATCGCTCTGTGGATGACTGAGTAC	24	134
rat-BCL-2-R	AGAGACAGCCAGGAGAAATCAAAC	24	
rat-*GAPDH*-F	AAGTTCAACGGCACAGTCAAGG	22	121
rat-*GAPDH*-R	CATACTCAGCACCAGCATCACC	22	

*Data analysis*

To draw standard curves the assay was performed on two-fold dilution series of the template DNA (*e.g*., 50, 25, 12.5, 6.25 and 3.13 ng).Then, the standard curves were drawn by plotting threshold cycles (CT) versus log DNA concentration. Amplification efficiency (E) of BCL-2, GAPDH genes was calculated according to following formula,

$E=\left[\left(10^{\frac{-1}{\mathrm{slope}}}\right)-1\right]\times 100$

The CT parameter was defined as the cycle number at which the amplification curve crossed a fixed threshold line**.**

Quantitative analysis was performed by the measurement of threshold cycle (CT) values during the exponential phase of amplification. The parameter CT was defined as the cycle number at which the amplification plot passed a fixed threshold. In each assay, mCT was the mean CT value of duplicate amplifications. Relative quantity of BCL-2 gene was determined using comparative CT method and ΔCT was calculated as the difference between the CT values of the BCL-2 and the CT value of *GAPDH* gene. The data were analyzed using the following formula: Gene dosage ratio = 2^−^^ΔΔCT^, where −ΔΔCT = [mCTBCL-2 (test sample) – mCT*GAPDH *gene (test sample) - [mCTBCL-2 (normal sample) −mCT*GAPDH* gene (normal sample)]. Gene dosage ratios were relative to the mean ΔCT value of these samples. Data processing was analyzed with ABI Prism 7300 Sequence Detection System (version 1.2.3, Applied Biosystems, UK). The graph preparation were performed using Microsoft Excel 2007 and RJS Graph 3.90.10 (20).

*Statical analysis*

Results were presented as mean ± standard deviation. Difference between groups with unilateral variance analysis test (ANOVA) and follow up Tukey test was estimated. P < 0.05 was used as the minimum level of significance mean’s differences. Quantitative evaluation of apoptotic cells in 5 microscopic fields, randomly the number of apoptotic cells determined and their means expressed as the ratio of apoptotic cells to total cells and to analyze the results of the four groups unilateral ANOVA test was used if any, to determine the groups which differed Turkey test was used. The data analyzed by SPSS-13.0 software. 

## Results

**Figure 1:**
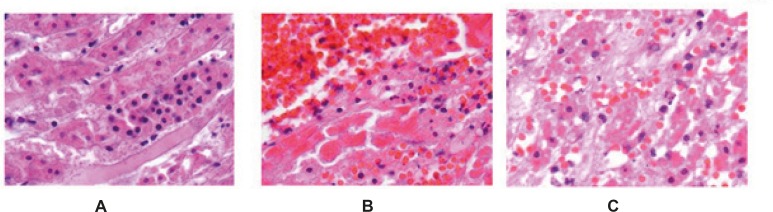
Microscopic view of the rat kidney tissue from control group that ischemia-reperfusion has been applied to them (a,b,c). Pay attention to the damaged cells with piknotic and kariocetic nucleus. (Hematoxylin-eosin staining and x40 magnification).

**Figure 2:**
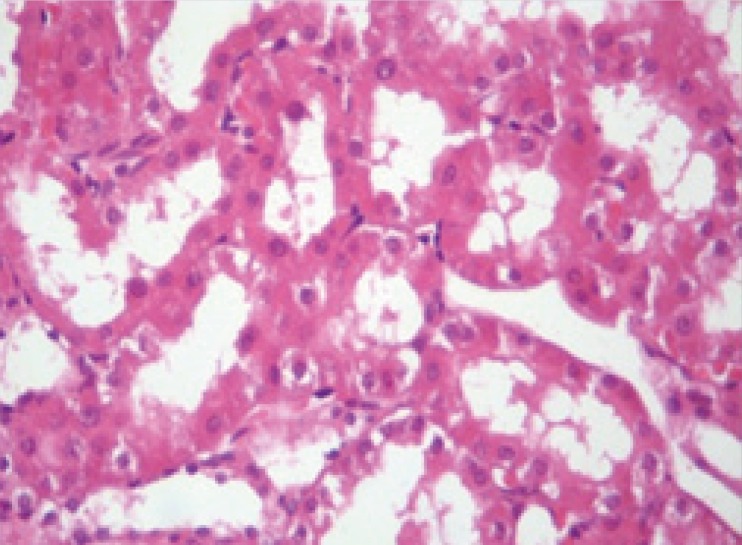
Microscopic view of the rat kidney tissue from treatment group with pantoxifylline (45 mg/Kg). Mild damage is visible in comparison with control group. (Hematoxylin-eosin staining and x40 magnification).

In the control group, ischemia-reperfusion caused severe changes of apoptosis in cells of renal tubules. In TUNEL staining, apoptotic cells were visible dark to light brown (Figures 1-2). Apoptosis rate in control group was 11.3 ± 0.62 and in treatment group with pantoxifylline was 0.34 ± 0.17 and showed a significant increase in control group (P < 0.001). 

**Figure 3:**
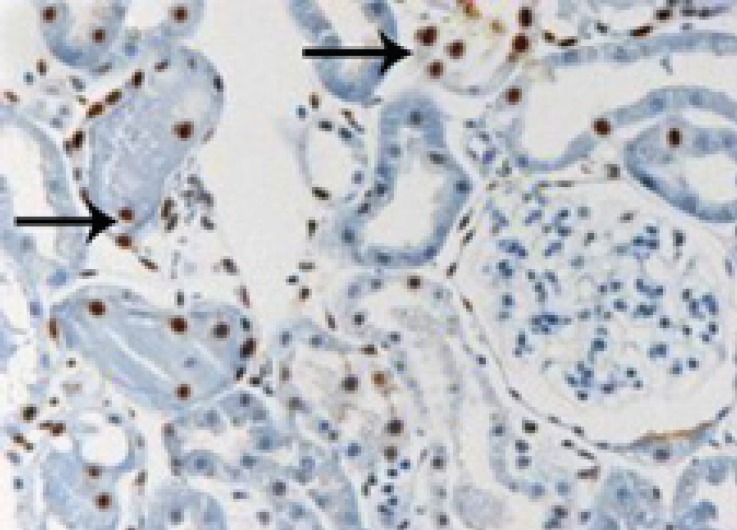
Microscopic view of the rat kidney tissue from control group that ischemia-reperfusion has been applied to them. Pay attention to the TUNEL-positive or apoptotic cells (arrows). (40×magnification).

**Figure 4:**
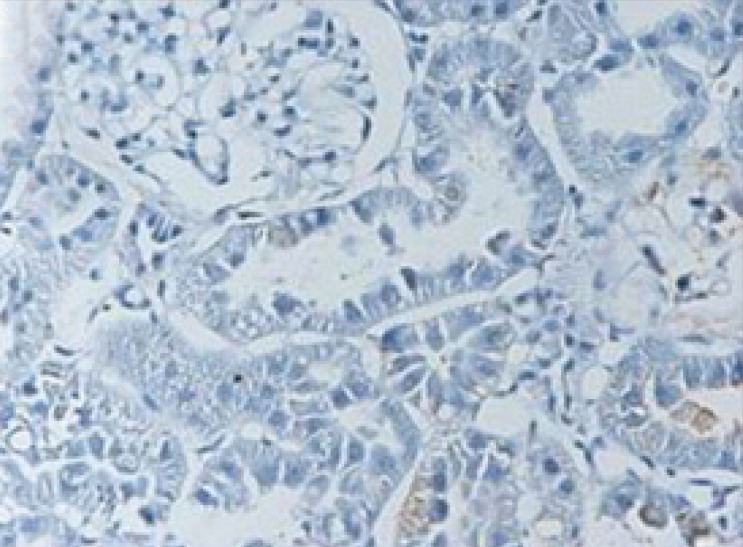
Microscopic view of the rat kidney tissue from treatment group with pantoxifylline (45 mg/Kg). Only a few TUNEL positive cells in comparison with control group are visible. (×40 magnification

In the group treated with pentoxifylline of 45 mg/Kg, the drug reduced apoptosis severity which caused by ischemia-reperfusion (Figures 3 and 4), that there was significant difference between this group (8.9 ± 0.35) and the control group (11.3 ± 0.62) (P < 0.05). 

**Figure 5:**
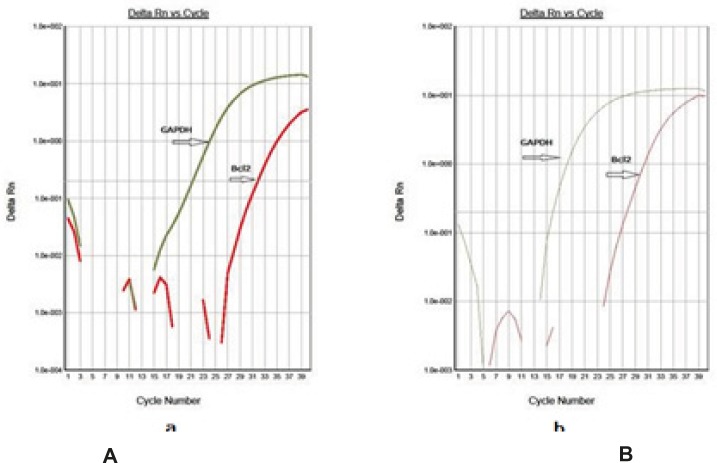
Amplification analysis of real-time PCR for BCL-2 and *GAPDH* genes. a: control group that ischemia-reperfusion, b:Treatment group with pantoxifylline

Using this method, tested and normal samples were analyzed. As expected, there was a significant difference between the tested and normal samples BCL-2 gene expression changes. To optimize and validate the real-time PCR assay before using ΔΔCT method for gene expression, a validation experiment was performed to determine the PCR efficiencies of the target and the reference genes. The input amount of template DNA was plotted against the corresponding CT values. The slope of the best-fit lines were within the acceptable range of −3.6 < slope < −3.1 (http://www.gene-quantification.de). The consistency of all the PCR reactions through a wide range of template DNA concentrations (3–50 ng) was assessed by plotting ΔCT values of BCL-2 gene against the input amount of DNA. The absolute slopes of the best-fit lines were ≤ 0.1 for BCL-2 gene which indicated the validity of the ΔΔCT relative quantitation. Melting curve analysis was performed for every single reaction to exclude amplification of non-specific products. Each valid amplification reaction displayed a single peak at expected Tm. Furthermore, gel electrophoresis analysis of the PCR products revealed a single band with the expected size for each amplicon .The results also showed the BCL-2 gene expression was declined in control group as compared to experimental groups (Figure 5). The target/reference (BCL-2/GAPDH) mean ratio was 0.58 ± 0.16 for control group and 1.14 ± 0.13 for treatment group.Therefore, this study was undertaken to reveal the effect of the pentoxifylline on BCL-2 gene expression changes in kidney after a period of ischemia or lack of oxygen in rats. Here, we designed and optimized quantitative real-time PCR assays using SYBR Green I technologies from Applied Biosystems.

## Discussion

One of the important issues in ischemia – reperfusion is cellular injury. Reperfusion induced contradictory cell injury in the tissue. Therefore, in addition to the cells that were irreversibly damaged until the end of ischemia, other cells in the tissue are destroyed (21). In this study the role of pentoxifylline was evaluated in cell injury and its significant results is reducing of cell death in the group treated with pentoxyfylline. In this study, warm ischemia time was 60 minutes because in a preliminary study that Hidehisa and his colleagues did in 2002, it was found that in rats were studied, 90 minutes ischemia due to kidney failure and will cause animal death and the animal is not able to tolerate 90 minutes of ischemia. In this study also, warm ischemia time was 60 minutes according to researchers’ study which this time caused sufficient ischemia-reperfusion injury in kidney (22). Kelly and colleagues in 1996, Daem and colleagues in 1999 concluded that the most damaged kidney function is in 24 or 48 hours after reperfusion (23, 24). Basile and colleagues in 1997 reported that the first occurrence of apoptosis induced by ischemia-reperfusion kidney injury which was notable in third day (25). 

Hidehisa and colleagues in 2002 expressed that in the control group 72 hours after ischemia by Doppler, superficial renal parenchyma’s blood flow decreased to 70 percent of the surface before ischemia but in the FR167653 group blood flow decreases less. Interleukin-1 and tumor necrosis factor is likely to be the main factors for thrombosis in small blood vessels and DIC. Because the FR167653 drug is the inhibitor of the interleukin-1 production and tumor necrosis factor. As a result,Hidehisa and colleagues assumed that the FR167653 drug because of its inhibitory efects on the interleukin-1 production and tumor necrosis factor, may improve the blood flow in kidney. In the present study the pantoxifylline drug also by reducing cytokine interleukin -1 production and tumor necrosis factor as well as its role in increasing Erythrocyte flexibility, decreasing blood viscosity and improvement of capillary circulation and having fibrinolyticproperty, maybe involved in improving renal blood flow and reduction of renal injury caused by ischemia-reperfusion that more studies and researches are needed in this field. Interleukin-1 and tumor necrosis factor are the famous inflammatory cytokine related to nephritis caused by autoimmune lupus and ischemia-reperfusion injury. In the ischemia-reperfusion, interleukin-1 and tumor necrosis factor released from resident macrophages in kidney and parenchyma renal cells can cause renal parenchymal damage by apoptosis and using neutrophils (that cause the release of oxygen metabolites and active proteases). Renal cells apoptosis and using neutrophils as the main form of death is related to renal ischemia-reperfusion injury .In addition,tumor necrosis factor by connecting to tumor necrosis factor receptor type1 or Fas ligand and looking forward the activation of intracellular endonucleases, will induce apoptosis in epithelial renal cells (22,26,27). Therefore, is concluded that inhibitory effect of pentoxifylline against tumor necrosis factor may play a role in reduction of impairment in renal function by inhibiting cell death of apoptosis form. Zabel study and colleagues in 1991 exhibited that the pantoxifylline drug via reducing superoxide production by neutrophils, significantly reduce the cell death. Free radical agents or superoxide induce cell death through different ways that the most important agents are in the cell membrane and mitochondria. It is notice worthy that the pantoxifyllinedrug by reducing oxidative stresses significantly reduces the cell death (28). 

Another agent to induce the cell death after ischemia-reperfusion is cytokines the tumor necrosis factor that by connecting to its specific receptor TNFR, induce cell death from the extracellular pathway. About the subject “the role of tumor necrosis factor in ischemic reperfusion injury", Koenig and colleagues in 2006 have done the study and showed that pentoxifylline significantly reduces the production of tumor necrosis factor in rheumatoid arthritis patients. It is notice worthy that this pantoxifylline’s function can inhibit the cell death; moreover,is effective in reducing inflammatory responses (29). 

Savic and colleagues have done the study on protective effect of pentoxifylline therapy in patients with acute renal failure and concluded that pentoxifylline by inhibiting expression of protein related to tumor necrosis factor, can reduce cell death (30). All comments that represent the results of this study are consistent with other studies in this field. In the process of reperfusion, inflammatory cytokines such as interleukin-1 and tumor necrosis factor cause the appearance of connective molecules in the surface of endothelium ischemic vessels’ tissue. ICAM-1 is one of the important connective molecules that tumor necrosis factor increases the expression and exhibition of this connective molecule in the surface of the endothelial cells, which finally lead to entrance of inflammatory cells particularly multicore cells to ischemic tissue and results in tissue damage. Production of myeloperoxidase from neutrophils and the reaction between NO and superoxide radical-induced inflammatory cells’ activity, causes production of proxy nitrite and the addition of oxidative stress which also causes the necrosis of tubular cells. Since pentoxifylline can inhibit inflammatory cytokine production (interleukin-1 and tumor necrosis factor); therefore, it reduces the inflammatory response and also can inhibit cell injury indirectly, which have been demonstrated in the results of microscopic studies the cell death process in treatment group. Frank and colleagues in 2004 showed that following the ischemia - reperfusion, other agents such as growth factors, edematous cytokines and various biochemical factors are released which induced cell death (32). One of these agents is the tumor factor necrosis which induces cell death of renal tubules after ischemia-reperfusion by two methods: 1-of the C cytochrome, 2-NF-KB. The tumor necrosis factor with positive effects on C cytochrome will activate Apaf-1 pathway and caspase 9 by activating caspase 3 results in cell death. Nuclear factor B play crucial role in transcription of main cell death genes and induce the tumor necrosis factor to activate and express the main cell death proteins .Therefore, by analyzing molecular-cellular pathways, role of tumor necrosis factor and cell death, inhibitory effect of pentoxifylline are obvious for results of this study (31).

We selected BCL-2 gene, because BCL-2 family of proteins functions as pro- and anti-apoptotic members (32). BCL-2 members such as *bax, bak, bad or bcl-Xs* promote apoptosis, whereas other members such as BCL*-*2 and *bcl-Xl* prevent apoptosis by blocking the translocation of cytochrome c, and subsequent caspase activation. Mitochondria are involved inexcitotoxic injury during cerebral ischemia and the release of cytochrome *c*, an apoptogenic factor that propagates death signals by triggering caspases leading to cell death. Using these assay, status of all subjects was successfully determined. We expanded the coverage of the detectable BCL-2 gene by SYBR Green assay for BCL-2 gene expression. This issue could be increased in the drug group and decressed in ischemia group. Cobe *et al*. indicated that in ischemia group the BCL-2 gene was decressed(33).Chien *et al* indicated that, as in many organs, the changed BCL-2 family proteins levels could contribute to apoptotic cell death in I/R kidneys (34).In the present study, the mean value of the ratios, obtained from tested and normal samples using SYBR Green assay for BCL-2 gene, was in agreement with the results reported by Chien*et et al*. (34), Cobe *et al.* (33) ,Khazaie *et al* (35) and Sari *et al. *(36). This study suggests that the drug pentoxifylline reduces renal ischemia-reperfusion injury and may be useful as a therapeutic drug in reducing delaying effect of connective tissue. Nowadays organ transplantation and rejection due to the occurrence of edematous reactions and cell injury are one of the most important topics and pantoxifylline drug as a phosphodiesterase inhibitor can be used in cases of kidney transplantation and significantly reduces production of IL- 10,IL- 6,IL- 1,TNF –α and prevents the rejection in renal transplant patients (28). It is suggested that further studies be started for the use of this drug in cases of organ transplantation till open the other new researches in this field. 
